# Adults remember their birth weight, an important anamnestic item for cardiovascular and renal disease risk stratification

**DOI:** 10.3389/fcvm.2022.1007524

**Published:** 2022-10-06

**Authors:** Alexandra Crivelli-Meyer, Olivier Giannini, Mario Giovanni Bianchetti, Giacomo Domenico Simonetti

**Affiliations:** ^1^Institute of Pediatrics of Southern Switzerland, Ente Ospedaliero Cantonale (EOC), Bellinzona, Switzerland; ^2^Faculty of Biomedical Sciences, Università della Svizzera Italiana, Lugano, Switzerland; ^3^Department of Medicine, Ente Ospedaliero Cantonale (EOC), Bellinzona, Switzerland

**Keywords:** birth weight, cardiovascular risk, Barker’s hypothesis, renal disease, low birth weight

## Abstract

To assess how many adults remember their own birth weight, an important anamnestic item for cardiovascular and renal disease risk stratification, we conducted an inquiry among all employees of public hospitals of Ente Ospedaliero Cantonale (EOC) in Ticino region (Southern Switzerland). The results show that the vast majority of adults remember their own birth weight. Hence, it is reasonable to include this information in the stratification of risk for cardiovascular and renal diseases.

## Introduction

Low birth weight confers an increased risk for cardiovascular and renal diseases (1). Consequently, it has been recommended that infants with growth-restricted, preterm, or low birth weight should undergo annual blood pressure measurement and urinalysis from the age of 3 years (2).

Like hypertension, physical inactivity, excessive body weight, smoking, diabetes, abnormal lipids and a positive family history of premature cardiovascular disease, low birth weight is a also relevant cardiovascular risk factor in adulthood (1, 3). Nevertheless, low birth weight is neglected in everyday clinical practice, at least partly because physicians assume that it is a feature that is unlikely to be remembered in adulthood.

## Materials and methods

To assess how many adults remember their own birth weight, all (about 5,000; 70% women) employees of public hospitals of Ente Ospedaliero Cantonale (EOC) in Ticino region (Southern Switzerland) were invited to fill in a close-ended web-based questionnaire addressing, among others, own weight at birth. Since the analysis was completely anonymous, the institutional review board waived the necessity to have approval for this type of study. In an effort to get as much information as possible, we analyzed the responses to 12 questions: 6 questions about demographic data of the participants, 3 questions about knowledge of birth weight, birth length, and duration of pregnancy, 2 questions about cardiovascular risk factors and diseases and their pharmacotherapy, and one question to assess how the participants get the birth information.

Low birth weight was defined as weight of less than 2,500 g. Continuous data are presented as median values with interquartile range or minimum and maximum range, and categorical data are presented as frequencies and percentages calculated from single answers. A comparison of prevalence between groups was performed by Fisher exact test. Mann-Whitney *U*-test was conducted to compare the two groups. All statistical analyses were performed using the GraphPad Prism^®^ software version 8.0 (GraphPad Software, San Diego, CA, United States). Significance was defined at *p* < 0.05.

## Results

A total of 1,369 (27%) employees answered the questionnaire, 1,054 (77%) were female and 315 (23%) were male. They ranged in the ages of 17 to 67 years (median 40 years), 677 (49%) were younger than 40 years, and 692 (51%) were 40 years of age or older. Twenty-one (1%) had an educational level of secondary school or less, 682 (54%) had higher professional education, and 567 (45%) had an university degree. but for 99 participants the educational level was not known. The profile of the employees who answered was similar to that of all employees (i.e., sex and educational level). A total of 1164 (85%) declared to remember their own birth weight, the majority (70%) of them knew this fact by heart, 24% asked their parents, and 6% searched this information on birth documents at home. The knowledge of birth weight was slightly but significantly lower in the subjects 40 years of age or older [572 out of 692 (83%) vs. 593 out of 677 (88%); *p* < 0.01]. Median declared birth weight was 3,300 g (interquartile range between 2,850 and 3,600 g). Low birth weight was present in 8.2% of the subjects, and 13% of the subjects presented at least one cardiovascular risk factor or disease. A trend toward an association between low birth weight and higher body mass index or cardiovascular diseases was found in this cohort (*p* = 0.16).

## Discussion

The majority of the adults remember their own birth weight, but the knowledge of it seems to be slightly lower in the elderly subjects. These conclusions might be flawed by some limitations. Given the rather low response rate (27%), our results should be considered preliminary and further confirmation is needed. Nevertheless, the educational level of the employees who answered is similar to that of all employees. Also, the high percentage of women who answered is in line to the high percentage of women working in public hospitals of Ente Ospedaliero Cantonale (EOC) in Ticino, Switzerland.

A possible bias of the results is the fact that the survey was carried out among hospital workers, and many of them are health professionals who generally have more knowledge of health history than the general population and that it is impossible to know the veracity of the data given in the survey by the participants since the answers for birth weight knowledge could not be confirmed with data from healthcare records. However, the birth weight distribution and mean values are in line with the reference birth weight percentiles (4).

Although further studies conducted on the general population are needed to confirm the preliminary results, we can assume that in developed countries such as Switzerland birth weight is correctly remembered, as a good agreement between birth record and self-reported birth weight is normally observed (5). Hence, it seems reasonable to include this information in the history for stratification of risk for cardiovascular and renal diseases not only in young patients but also in patients older than 40 years of age.

## Data availability statement

The raw data supporting the conclusions of this article will be made available by the authors, without undue reservation.

## Ethics statement

The studies involving human participants were reviewed and approved by the Ethics Committee of the Canton of Ticino.

## Author contributions

AC-M: study design, collection of data, analysis of data, and first draft of the manuscript. OG: study design, analysis of data, and final draft of the manuscript. MB: study design and final draft of the manuscript. GS: study design, collection of data, analysis of data, and final draft of the manuscript. All authors contributed to the article and approved the submitted version.
